# Infectious Bronchitis Virus Nonstructural Protein 4 Alone Induces Membrane Pairing

**DOI:** 10.3390/v10090477

**Published:** 2018-09-06

**Authors:** Nicole Doyle, Benjamin W. Neuman, Jennifer Simpson, Philippa C. Hawes, Judith Mantell, Paul Verkade, Hasan Alrashedi, Helena J. Maier

**Affiliations:** 1 The Pirbright Institute, Pirbright, Surrey GU24 0NF, UK; nicole.doyle@pirbright.ac.uk (N.D.); jennifer.simpson@pirbright.ac.uk (J.S.); pippa.hawes@pirbright.ac.uk (P.C.H.); 2 School of Biological Sciences, University of Reading, Reading RG6 6AJ, UK; bneuman@tamut.edu (B.W.N.); h.s.h.alrashedi@student.reading.ac.uk (H.A.); 3 School of Biochemistry, University of Bristol, Bristol BS8 1TD, UK; j.mantell@bristol.ac.uk (J.M.); p.verkade@bristol.ac.uk (P.V.)

**Keywords:** coronavirus, infectious bronchitis virus, non-structural protein, nsp4, nsp3, membrane rearrangements, electron tomography, paired membranes, zippered ER

## Abstract

Positive-strand RNA viruses, such as coronaviruses, induce cellular membrane rearrangements during replication to form replication organelles allowing for efficient viral RNA synthesis. Infectious bronchitis virus (IBV), a pathogenic avian *Gammacoronavirus* of significant importance to the global poultry industry, has been shown to induce the formation of double membrane vesicles (DMVs), zippered endoplasmic reticulum (zER) and tethered vesicles, known as spherules. These membrane rearrangements are virally induced; however, it remains unclear which viral proteins are responsible. In this study, membrane rearrangements induced when expressing viral non-structural proteins (nsps) from two different strains of IBV were compared. Three non-structural transmembrane proteins, nsp3, nsp4, and nsp6, were expressed in cells singularly or in combination and the effects on cellular membranes investigated using electron microscopy and electron tomography. In contrast to previously studied coronaviruses, IBV nsp4 alone is necessary and sufficient to induce membrane pairing; however, expression of the transmembrane proteins together was not sufficient to fully recapitulate DMVs. This indicates that although nsp4 is able to singularly induce membrane pairing, further viral or host factors are required in order to fully assemble IBV replicative structures. This study highlights further differences in the mechanism of membrane rearrangements between members of the coronavirus family.

## 1. Introduction

Viruses rely on their host cell to provide most of what they need to replicate and in order to do this, they hijack many cellular processes. A well-studied example is the ability of positive-sense single-stranded RNA viruses (+RNA) to induce cellular membrane rearrangements upon expression of viral proteins [1,2]. This reorganization of cellular membranes is a critical step in the viral replication cycle since these areas of restructured membranes act as a site for assembly of all components required for viral RNA synthesis as well as offer protection from detection by the host antiviral defenses [3,4]. Although the structures of these membranes are relatively well-understood, the mechanisms behind their formation, and particularly the viral and host proteins involved, are often not.

The precise structure of virally induced membrane rearrangements varies between viruses [5,6], but viruses generally cause proliferation of membranes, forming structures, such as convoluted membranes (CM), as well as distinct types of vesicles. Most common are double membrane vesicles (DMVs), which are discrete from the cytoplasm and are produced by viruses, such as poliovirus [7,8], hepatitis C virus [9,10], human norovirus [11], and recently the equine torovirus, Berne virus [12]. Spherules, which are invaginated vesicles with a channel connecting them to the cytoplasm, have been found in Semliki Forest virus [13], some *Flaviviruses* [14,15,16,17], as well as Brome mosaic virus (BMV), which is able to induce their formation with the expression of just one viral protein [18].

An important +RNA virus family, the coronaviruses, include pathogens of both animal and human importance, such as severe acute respiratory syndrome coronavirus (SARS-CoV), Middle East respiratory syndrome coronavirus (MERS-CoV), mouse hepatitis virus (MHV), porcine epidemic diarrhea virus (PEDV), and infectious bronchitis virus (IBV). Within this subfamily of viruses, we see variations in membrane rearrangements formed. DMVs and CM are found in cells infected with the *Alpha*- and *Betacoronaviruses*, such as SARS-CoV, MERS-CoV, and MHV [19,20,21,22,23,24]. In the case of the *Gammacoronavirus* IBV, although DMVs are found, the virus induces little CM and instead induces membrane zippering to form zippered endoplasmic reticulum (zER) as well as double membrane spherules, which are found tethered to the zER [25], producing a much more defined structure when compared to CM. Subsequent to this discovery, MERS-CoV infection has also been shown to produce small circular structures similar in appearance to the spherules seen in IBV infection but less distinct [26].

The coronaviral proteins involved in the production of membrane rearrangements have been recently investigated with the three transmembrane non-structural proteins (nsps) nsp3, 4, and 6, which are the focus of these studies. Nsps 3, 4, and 6 from different coronaviruses are accepted as functional homologues, although amino acid sequence conservation is low (ranging from 13.4 to 25.9% amino acid homology for nsps 3, 4, and 6 between IBV strain BeauR and MHV strain A59). These proteins do, however, have conserved secondary structure and conserved domains, including enzymatic domains in nsp3, transmembrane domains in all three proteins, and cytoplasmic endo-domains in nsps 4 and 6. For a detailed review of the domain organization and known functions of nsps 3, 4, and 6, see [27]. Nsp4 of MHV has been shown to be important for the normal function and stability of DMVs, where mutations in nsp4 resulted in attenuated virus and impairment of DMV formation [28,29,30]. In addition, nsp3 has been shown to localize to DMVs and CM in SARS-CoV-infected cells [31]. In a related group of viruses, the *Arteriviruses*, expression of two nsps (nsps2 and 3) was able to produce DMVs [32,33,34]. These nsps of the arterivirus are considered functional homologs to coronavirus nsp3 and 4 [35]. Upon co-expression of nsp3 and 4 from MHV, both proteins located to areas of curved membranes from where they were shown to be able to recruit nsp2 and 6; however, nsp3 and 4 alone were not able to induce the formation of DMVs [36,37]. Following on from this, it was shown that co-expression of SARS-CoV nsp3 and 4 induced membrane pairing and with the addition of nsp6 the formation of DMV-like structures [38]. In a subsequent study by others, it was shown that expression of only nsp3 and 4 from either MERS-CoV or SARS-CoV was able to induce DMV formation, and furthermore, addition of nsp6 made no difference to their shape or size, and did not induce the spherule-like structures seen following infection with whole virus [26]. Interestingly, however, a small molecule inhibitor, K22, has been shown to inhibit the replication of several coronaviruses in vitro. In HCoV-229E, K22 impaired DMV formation, while K22 resistance was associated with mutations in nsp6, emphasizing a role for nsp6 in DMV formation [39].

IBV is a pathogen of poultry, causing significant economic losses to the poultry industry worldwide as well as animal welfare problems. Various strains of IBV cause disease that varies in severity from mild respiratory problems to virulent strains that can cause nephropathology and reproductive organ pathology. In this study, we compared the membrane rearrangements induced by viral proteins from two different strains of IBV, the pathogenic M41 and the apathogenic BeauR. These strains were chosen because BeauR and other strains of IBV induce DMV, zER, and spherule formation; however, M41 produces a low spherule phenotype when compared with other strains of the virus [40]. As the role in membrane rearrangements for nsp3 and 4 is well-established for several nidoviruses and considering that nsp6 may also play some role, here we investigated the role that these three nsps play in the formation of IBV membrane rearrangements.

## 2. Materials and Methods

### 2.1. Cells, Viruses, and Plasmids

Avian DF1 cells were maintained in DMEM (Sigma Aldrich, Gillingham, UK) supplemented with 10% FCS (Sigma Aldrich, Gillingham, UK). IBV strains BeauR and M41-CK (here referred to as M41) have been described previously [41,42]. Plasmids expressing tagged nsps derived from either the apathogenic strain BeauR or the pathogenic strain M41 were generated to produce pEGFP-N1-M41 nsp3, pmCherry-N1-BeauR nsp4, pmCherry-N1-M41 nsp4, pcDNA3.1(-)-BeauR nsp6-3xFLAG, and pcDNA3.1(-)-M41 nsp6-3xFLAG. RNA was extracted from virus-infected cells using an RNAeasy kit (Qiagen, Hilden, Germany) following the manufacturer’s protocol. RNA was reverse transcribed using Superscript III (Fisher Scientific, Loughborough, UK) and a random primer following the manufacturer’s protocol. PCR was carried out on cDNA using primers specific for each gene, including flanking restriction sites. PCR products were digested and ligated into pEGFP-N1 (Takara Bio Europe, Saint-Germain-en-Laye, France) or pmCherry-N1 (Takara Bio) using XhoI and BamHI restriction sites. Plasmid pcDNA3.1(-) was modified by insertion of a 3xFLAG motif between the KpnI and HindIII sites to generate pcDNA3.1(-)-3xFLAG. The PCR products were then ligated into this backbone using the XhoI and BamHI restriction sites. Plasmid sequences were verified using Sanger sequencing. The ER marker plasmid pYFP-ER was kindly provided by Dalan Bailey.

### 2.2. SDS-PAGE and Western Blotting

DF1 cells seeded into six-well plates were transfected with pEGFP-N1-M41 nsp3, pmCherry-N1-BeauR nsp4, pmCherry-N1-M41 nsp4, pcDNA3.1(-)-BeauR nsp6-3xFLAG or pcDNA3.1(-)-M41 nsp6-3xFLAG, pEGFP-C2, pmChery-N1, or pcDNA3.1(-)-BeauR nsp7-3xFLAG using lipofectamine 2000 (Fisher Scientific). Cells were transfected with a total of 1000 ng plasmid with a DNA:lipofectamine 2000 ratio of 1:3 following the manufacturer’s instructions. After 24 h, cells were lysed in cell lysis buffer (25 mM Tris-HCl (pH 7.4), 150 mM NaCl, 1 mM EDTA, 1% *v*/*v* Triton-X100, 5% *v*/*v* glycerol, 1× HALT protease inhibitor complex (Fisher Scientific). Cell lysates were heated with 4× sample buffer (Bio-rad Laboratories, Watford, UK) and separated on 4–20% TGX gels (Bio-rad). Proteins were transferred to a nitrocellulose membrane and blocked in 5% milk in PBS-T. Membranes were incubated with primary antibodies to detect GFP (Biolegend, London, UK), mCherry (Abcam, Cambridge, UK), or FLAG (M2; Sigma Aldrich, Gillingham, UK). After 1 h, membranes were washed with PBS-T and incubated with IRDye conjugated secondary antibodies (LI-COR, Cambridge, UK). Membranes were imaged using an Odyssey CLx Infrared imaging system (LI-COR).

### 2.3. Immunofluorescence Labelling

DF1 cells seeded onto glass coverslips were transfected with pEGFP-N1-M41 nsp3, pmCherry-N1-BeauR nsp4, pmCherry-N1-M41 nsp4, pcDNA3.1(-)-BeauR nsp6-3xFLAG, and pcDNA3.1(-)-M41 nsp6-3xFLAG alone or in combination using lipofectamine 2000. Cells were transfected with a total of 500 ng plasmid with a DNA:lipofectamine 2000 ratio of 1:2 following the manufacturer’s instructions. After 24 h, cells were fixed for 20 min in 4% paraformaldehyde in PBS at room temperature. Cells were then permeabilized in 0.1% Triton X-100 in PBS for 10 min and blocked in 0.5% BSA in PBS for 1 h. Primary anti-FLAG M2 antibody (Sigma Aldrich) and anti-PDI antibody (Enzo Life Sciences, Exeter, UK) were diluted in blocking buffer and cells incubated for 1 h. After three washes in PBS, Alexa fluor conjugated secondary antibodies (Fisher Scientific) were diluted 1/500 and cells incubated for 1 h. After a further three washes in PBS, nuclei were strained using ToPro3 (Fisher Scientific) or DAPI (Sigma Aldrich) and coverslips mounted with Vectashield (Vector Laboratories, Peterborough, UK). Cells were visualized using a Leica SP5 confocal microscope (Leica Microsystems, Milton Keynes, UK). Quantitation of transfected cells was performed manually on three randomly selected fields of view.

### 2.4. Transmission Electron Microscopy

DF1 cells in six-well plates were either infected with BeauR and incubated for 1 h at 37 °C when fresh 1× BES medium (MEM, 0.3% tryptose phosphate broth, 0.2% bovine serum albumin, 20 mM *N*,*N*-Bis(2-hydroxyethyl)-2-aminoethanesulfonic acid (BES), 0.21% sodium bicarbonate, 2 mM l-glutamine, 250 U/mL nystatin, 100 U/mL penicillin, and 100 U/mL streptomycin) was added, or were transfected with plasmids as described above. At 24 hpi, cells were washed once in 0.9% saline and scraped into the saline buffer. Cells were pelleted at 500× *g* for 5 min at 4 °C and 500 μL 2% glutaraldehyde in 0.1 M sodium cacodylate was added to the pellet. DF1 cells were then rinsed three times in 0.1 M sodium cacodylate and incubated in 1% osmium tetroxide for 2 h. After three washes in water, cells were incubated in 2% uranium acetate aqueous for 2 h at 4 °C. Cells were dehydrated in increasing concentrations of acetone and then embedded in Agar 100 resin (Agar Scientific, Stansted, UK). Sections approximately 50 to 60 nm in thickness were cut and stained with 2% uranyl acetate to enhance contrast. Data was recorded at 80 kV on a Phillips CM20 (Amsterdam, Netherlands) with a charge-coupled device (CCD) camera. Cell sections used here each contained a single visible nucleus, with intact nuclear and plasma membranes. Alternatively, DF1 cells were seeded onto Thermanox coverslips (Fisher Scientific) and either infected with BeauR and incubated for 1 h at 37 °C, after which time fresh 1× BES medium was added, or cells were transfected with plasmids as described above. After 24 h, cells were fixed in 2% glutaraldehyde for 1 h, incubated in 1% aqueous osmium tetroxide solution for 1 h, then dehydrated in increasing concentrations of ethanol. Cells were embedded into Agar 100 resin and sections of 80 nm were cut, collected on hexagonal 200 thin bar copper grids, and stained with 2% uranyl acetate and lead citrate. Data was recorded on a FEI Tecnai 12 TEM (FEI, Cambridge, UK) used at 100 kV with a TVIPS F214 digital camera.

### 2.5. Electron Tomography

DF1 cells seeded onto Thermanox coverslips were transfected and processed as before. Sections 250 or 300 nm thick were cut from the resin-embedded blocks and collected on 50 mesh copper hexagonal grids coated in formvar or pioloform-coated copper slot grids. Ten or 15 nm gold particles were applied to the grids to serve as fiducial markers for subsequent alignments. Data was recorded on a JEOL 2100 F TEM (Jeol, Welwyn Garden City, UK) used at 200 kV with a TVIPS F416 digital camera, or on a Tecnai 20 TEM (FEI) used at 200 kV with a FEI 4 k × 4 k Eagle CCD camera. Samples were mounted in a JEOL high angle tilt holder or a Fischione double tilt tomography holder, respectively. A single axis tilt series was collected using Serial EM or FEI software. Each single axis tilt series was collected over 100° to 130° in increments of between 1° and 2.5° and subsequently aligned and reconstructed in IMOD [43].

## 3. Results

### 3.1. IBV Induces Typical Membrane Rearrangements in DF1 Cells

**Figure 1:**
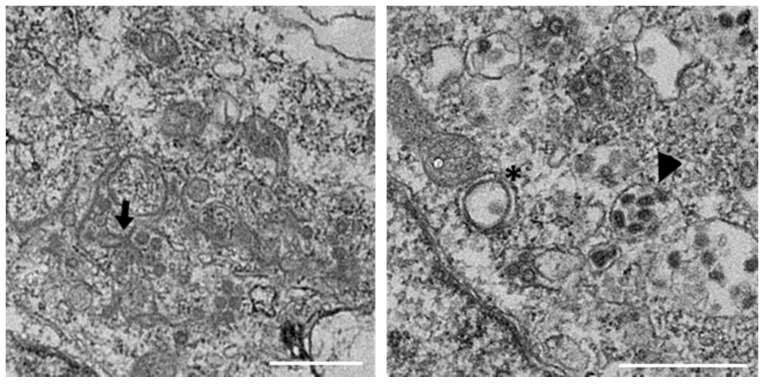
Membrane rearrangements in DF1 cells infected with infectious bronchitis virus (IBV) BeauR. DF1 cells were infected with BeauR for 24 h, fixed, and processed for electron microscopy (EM). Viral particles are indicated with arrowheads, double membrane vesicles (DMVs) with asterisks, and zippered endoplasmic reticulum (zER) and associated spherules with arrows. Scale bar represents 500 nm.

Our previous studies have shown that IBV is able to induce diverse membrane rearrangements in Vero cells, primary chicken kidney cells (CKCs) and tracheal organ cultures (TOCs). These membrane rearrangements include DMVs, zER, and spherules [25,40]. In order to further characterize membrane rearrangements induced by IBV, we analyzed the membrane rearrangements induced by BeauR in DF1s. Unlike primary CKCs, DF1s are a continuous avian cell line that are more easily transfected and are therefore used throughout this study. Although the spike protein of BeauR has increased tropism to allow for virus entry into additional cell lines, including DF1 cells, M41 is not adapted to infect these cells [44,45]. DF1 cells were infected with BeauR, fixed after 24 h, processed for EM, and imaged. Consistent with previous work, DMVs, zER, and spherules were all seen in IBV-infected DF1 cells (Figure 1).

### 3.2. Non-Structural Proteins 3, 4, and 6 Relocalize upon Co-Expression

**Figure 2:**
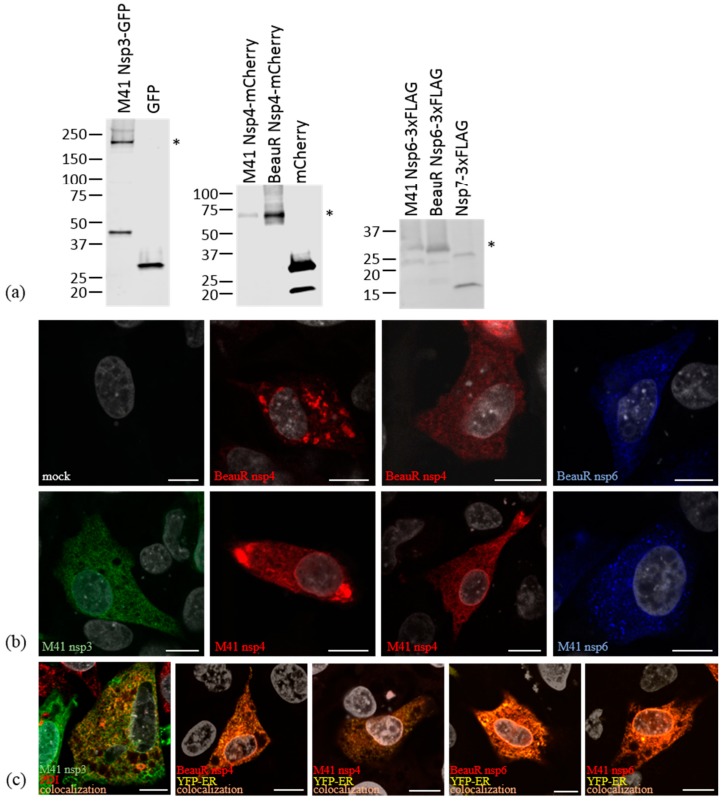
Expression of IBV non-structural proteins (nsps) in DF1 cells. (**a**) Detection of expression of viral nsps in transfected DF1 cell lysates. Cells were transfected with plasmids expressing tagged nsps, as indicated, or empty vectors or nsp7-3xFLAG as controls. Cell lysates were separated by SDS-PAGE and proteins detected by Western blot. From left to right, nsp3-GFP detected using anti-GFP, nsp4-mCherry detected using anti-mCherry, and nsp6-3xFLAG detected using anti-FLAG, as labelled. Molecular weight markers are shown on the left and asterisks indicate the nsp bands on each blot. (**b**) DF1 cells were transfected with plasmids expressing nsp4-mCherry and nsp6-3xFLAG from BeauR, and nsp3-EGFP, nsp4-mCherry, and nsp6-3xFLAG from M41. After 24 h, cells were fixed with 4% paraformaldehyde and imaged. Nsp3 (green), nsp4 (red), and nsp6 (blue) were imaged as labelled. Nuclei were stained with ToPro3 (grey) and scale bars indicate 10 μm. (**c**) DF1 cells were transfected with plasmids expressing nsp4-mCherry and nsp6-3xFLAG from BeauR, and nsp3-EGFP, nsp4-mCherry, and nsp6-3xFLAG from M41 together with YFP-ER. After 24 h, cells were fixed with 4% paraformaldehyde and imaged. Nsp3 (green) and nsp4 and nsp6 (red) were imaged along with markers for the ER; PDI (red) or YFP-ER (yellow) as indicated. Nuclei were stained with DAPI (grey) and scale bar represents 10 µm.

Other viruses in the *Nidovirales* order have been shown to require expression of only two or three nsps to induce membrane rearrangements similar to those seen under virus infection conditions [26,34,38]. To begin to understand the roles of IBV nsps in rearranging cellular membranes, nsps4 and 6 from apathogenic BeauR and nsps3, 4, and 6 from pathogenic M41 were tagged with fluorescent or epitope tags. It was not possible to generate a plasmid expressing nsp3 from BeauR due to presumed toxic sequences, as has been found for this region in other coronaviruses [46,47,48]. DF1 cells were transfected with these plasmids and after 24 h cells were lysed and proteins separated by SDS-PAGE and detected by Western blot. All fusions proteins were found to be intact with bands detectable at the predicted molecular weights (Figure 2a), although an additional 49 kDa band was present in nsp3-GFP expressing cells, presumably due to a cleavage event within nsp3. It was also noted that nsp6-3xFLAG from M41 migrated at a higher molecular weight than nsp6-3xFLAG from BeauR, most likely due to differences in post-translational modification. Subsequently, DF1 cells were transfected with these plasmids and after 24 h cells were fixed, labelled with an anti-FLAG antibody, and visualized by confocal microscopy. All three nsps showed reticular cytoplasmic labelling consistent with localization to the ER (Figure 2b), as has been observed previously [26,37,49,50,51,52,53]. In addition to ER localization, nsp4 was found in both small and large puncta in cells where the level of nsp4 expression was higher (comparison shown in Figure 2b). Nsp6 was also found in small cytoplasmic puncta when expressed alone (Figure 2b). To confirm ER localization, DF1 cells were transfected with either the plasmid expressing nsp3-GFP alone or plasmids expressing nsp4 or 6 together with pYFP-ER, as indicated. After 24 h, cells were fixed and labelled with anti-FLAG- and nsp3-expressing cells with anti-protein disulphide isomerase (PDI), a resident ER protein. Colocalization between YFP-ER or PDI and nsp3, 4, and 6 was observed, confirming that these proteins localize to the ER (Figure 2c).

**Figure 3:**
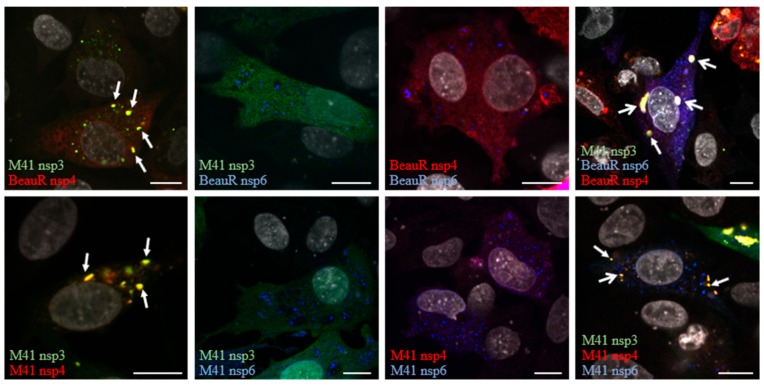
Co-expression of IBV non-structural proteins results in their relocalization from the ER to cytoplasmic foci. DF1 cells were transfected with plasmids expressing nsp4-mCherry and nsp6-3xFLAG from BeauR, and nsps3-EGFP, nsp4-mCherry, and nsp6-3xFLAG from M41 in pairs or in a combination of three, as indicated. Solid arrows indicate areas of nsp3 and 4 colocalization, open arrows indicate areas of nsp3, 4, and 6 colocalization. Nuclei were strained with ToPro3 (grey) and scale bar represents 10 µm.

Next, to understand whether co-expression of these proteins results in changes in their localization, DF1 cells were transfected with combinations of the plasmids. After 24 h, cells were fixed and labelled with an anti-FLAG antibody. Upon co-expression of some combinations of these viral proteins, this staining pattern changed. Expression of nsp3 with nsp4 resulted in both proteins localizing to cytoplasmic puncta, although some signals for both proteins also remained in the ER (Figure 3). Co-expression of nsp3 with nsp6, or nsp4 with nsp6, did not result in relocalization of either protein, with nsp3 remaining ER-associated, nsp4 remaining both ER-associated and localized in cytoplasmic puncta, and nsp6 remaining both ER-localized and in cytoplasmic puncta (Figure 3). Interestingly, co-expression of nsps3, 4, and 6 resulted in relocalization of all three proteins to cytoplasmic puncta, some containing nsp3 and 4, some nsp6 only, and some puncta containing nsp3, 4, and 6 (Figure 3). Nsps4 and 6 derived from either BeauR or M41 exhibited the same pattern of localization. This demonstrates that co-expression of IBV nsps in the absence of any other viral components can result in their relocalization within the cell, presumably as a result of protein–protein interactions and potentially associated with rearrangement of cellular membranes.

### 3.3. IBV nsp4 Alone Is Necessary and Sufficient to Induce Membrane Pairing

**Figure 4:**
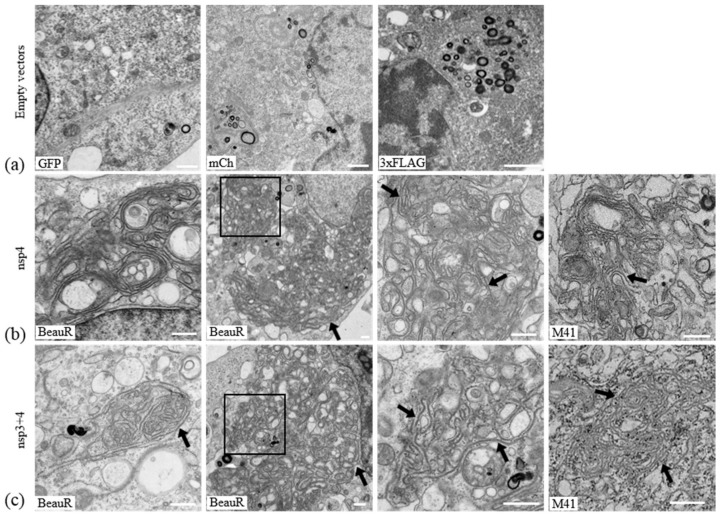
Expression of IBV nsp4 induces pairing of cellular membranes with no additional role for nsp3. DF1 cells were transfected with (**a**) the empty vectors: GFP, mCherry, and 3xFLAG; with a plasmid expressing BeauR or M41 nsp4-mCherry either (**b**) alone or (**c**) with a plasmid expressing M41 nsp3-EGFP, as indicated. After 24 h, cells were fixed and processed for EM. Areas of paired membranes are indicated by an arrow. The third image in (**b**,**c**) is a higher magnification of the boxed area in the second image. Scale bar represents 500 nm.

To further understand the ability of IBV nsps3, 4, and 6 to rearrange cellular membranes, proteins were expressed in cells and analyzed by electron microscopy (EM). Initially, to assist with subsequent analysis by EM, the percentage of total cells in Figure 2b and Figure 3 that were expressing the nsps of interest, as well as the percentage of cells expressing other combinations of nsps, was quantified (Table S1). DF1 cells were transfected with tagged nsp3, 4, and 6 derived from BeauR or M41 alone and in combination. After 24 h, cells were chemically fixed, embedded in resin, and visualized using an electron microscope. A phenotype common to all transfected cells was small, tight whorl-like structures which stained more strongly than other structures (Figure 4a). These were considered an artefact of transfection. Transfection of cells with empty pEGFP-N1, pmCherry-N1, or pcDNA3.1(-)-3xFLAG did not result in changes to cellular membranes (Figure 4a). Different types of membrane structures were observed in the transfected cell samples that were absent from mock treated cells, including paired membranes, disordered or piled membranes, and DMV-like structures. Nsp4 in other coronaviruses has been shown to be important in membrane modifications, particularly in the formation of conventional DMVs [29,30]. Initially, the effect of expression of nsp4 in DF1 cells was investigated. Interestingly, it was observed that expression of BeauR nsp4 alone was capable of forming paired membranes. This the first time this has been observed for any coronavirus nsp4. These paired membranes were observed both as very large areas of extensive accumulations or as small regions of shorter sections of paired membranes. The paired membranes were tightly apposed, often connected to the ER, were largely free of ribosomes, and strongly resembled IBV-induced zER (Figure 4b), although the electron density often surrounding IBV-induced zER was missing here and no spherules were present. Transfection of M41 nsp4 also induced membrane pairing (Figure 4b) with an appearance comparable to that of BeauR nsp4-induced paired membranes. For cells transfected with the BeauR nsp4 expression vector, 31 out of 235 cell sections (13%, percentage of total cells not transfected cells) contained piled membranes, and 3 out of 108 (3%) sections for M41, significant to *p* < 0.00001 by a Fisher’s exact test.

It has previously been shown for other coronaviruses that membrane pairing requires co-expression of nsps3 and 4 or that co-expression of these proteins results in DMV accumulation [26]. Therefore, the effect of co-expression of nsp3 with nsp4 was investigated. Firstly, the effect of expression of nsp3 alone on cellular membranes was determined. Although over 200 cells were examined from multiple experiments, expression of nsp3 was found to have no striking phenotype with cellular membranes appearing unchanged in the presence of nsp3 derived from M41 when compared with untransfected cells. Furthermore, surprisingly, expression of nsp3 with nsp4 had no effect on the membrane pairing ability of nsp4 (Figure 4c), with membrane rearrangements appearing comparable to cells expressing nsp4 alone, i.e., paired membranes connected to the ER and lacking ribosomes, found covering both large and smaller areas of the cytoplasm. Specifically, the numerous DMV-like structures observed in cells expressing nsp3 and 4 from either MERS-CoV or SARS-CoV were not observed here [26,38]. Overall, this data confirms that IBV nsp4 alone is the main driving factor in membrane pairing and co-expression of nsp3 does not alter this function.

### 3.4. Nsp6 Induces the Formation of Piled, Disordered Membranes

**Figure 5:**
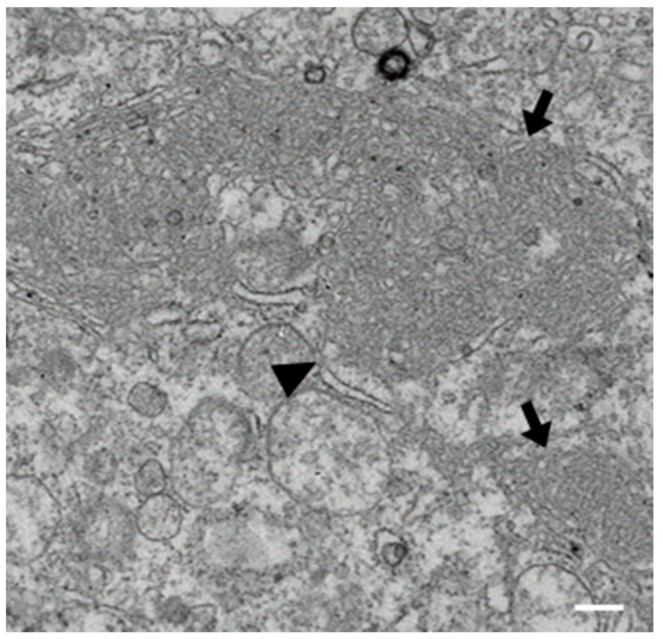
Expression of nsp6 alone induces the formation of piled, disordered membranes. DF1 cells were transfected with plasmids expressing nsp6-3xFLAG from BeauR. After 24 h, cells were fixed and processed for EM. Arrows indicate areas of piled, disordered membranes and the arrowhead indicates the area where piled membranes are derived from ER. Scale bar represents 500 nm.

Coronavirus nsp6 has previously been linked to autophagy induction when expressed alone [49]. Nsp6 derived from SARS-CoV has also been shown to induce single membrane vesicle accumulation and microtubule organizing center vesiculation [38]. Therefore, the cellular membrane rearrangements induced by expression of IBV nsp6 were analyzed. In cells expressing nsp6 alone from either BeauR or M41, large areas of tangled single membranes, which appear to be derived from the ER, were observed (Figure 5). These piled, disordered membranes strongly resemble the disordered membrane bodies seen previously upon expression of SARS-CoV nsp3 [38]. To determine the effect of expression of nsp6 with other nsps on the formation of disordered membranes or any other structures, samples transfected with plasmids expressing nsp6 and either nsp4 or nsp3 were analyzed. In cells co-expressing nsp3 and 6, it was observed that cells expressing BeauR nsp6 formed disordered membranes while those expressing M41 nsp6 did not. Co-expression of nsp4 and nsp6 produced the paired membranes associated with nsp4 expression (for both BeauR and M41 nsp4). Disordered membranes were only found in cells co-expressing BeauR nsp6 but none when co-expressing M41 nsp6. This indicates that while nsp6 from either BeauR or M41 can induce the formation of disordered membranes when expressed singly, co-expression of nsp6 with either nsp3 or 4 disrupts this mechanism and to a greater extent in M41.

### 3.5. Nsps 3, 4, and 6 Are Not Able to Recapitulate the IBV Replication Organelle

**Figure 6:**
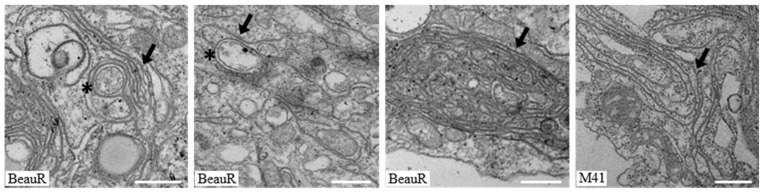
A limited number of DMV-like structures are formed in cells transfected with IBV nsp3, 4, and 6. DF1 cells were transfected with M41 nsp3 and either BeauR nsp4 and 6 or M41 nsp4 and 6. After 24 h, cells were fixed and processed for EM. Paired membranes are indicated with an arrow, DMV-like structures indicated with an asterisk. Scale bar represents 500 nm.

Finally, the membrane rearrangements induced by co-expression of IBV nsps3, 4, and 6 were investigated by electron microscopy to determine whether co-expression of all three transmembrane nsps could result in the formation of structures comparable to replication organelles in IBV-infected cells. The major phenotype observed following co-expression of all three nsps was the paired membranes induced by expression of nsp4 alone (Figure 6). When nsp4 and nsp6 derived from BeauR were expressed with M41 nsp3, a very limited number of DMV-like structures was observed (3 in 329 cell sections). In cells co-expressing nsp3, 4, and 6 derived from M41, no DMV-like vesicles were found in 489 cell sections with only nsp4-associated paired membranes being detected. In neither combination were the spherules usually found during virus infection observed. Therefore, although co-expression of IBV nsps 3, 4, and 6 may be sufficient for formation of DMVs, this does not seem to be a very efficient process compared with DMV formation by nsp3 and 4 from the *Betacoronaviruses* studied previously [26,38] and nsp6 is unlikely to be the additional nsp required for IBV DMV formation.

### 3.6. IBV nsp3 and 4 Do Not Induce DMVs

**Figure 7:**
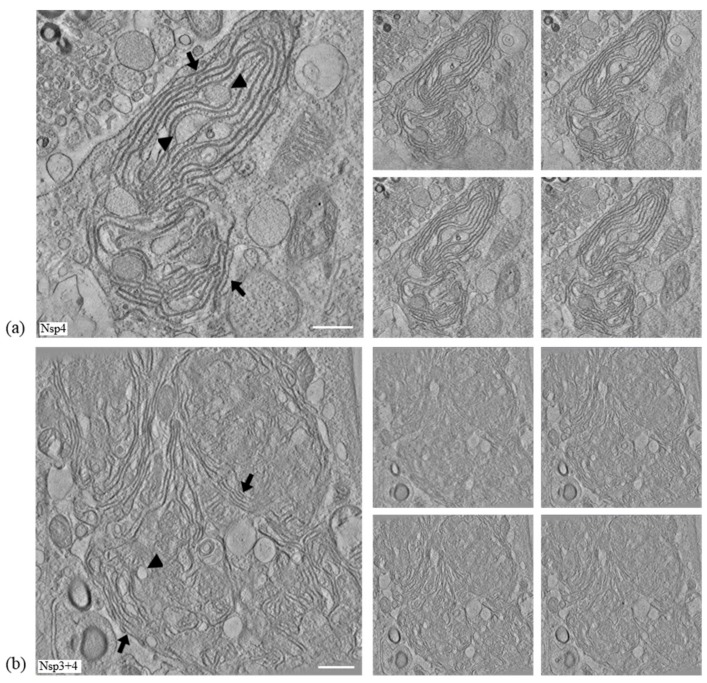
Expression of IBV nsp4 induces the formation of sheet-like areas of paired membranes with no additional role for nsp3. DF1 cells were transfected with a plasmid expressing BeauR nsp4-mCherry either (**a**) alone or (**b**) with a plasmid expressing M41 nsp3-EGFP, as indicated. After 24 h, cells were fixed and processed for ET, an average of five slices as well as four individual slices is shown here. Areas of paired membranes are indicated by arrows, areas where paired membranes dilate are indicated by arrowheads. Scale bar represents 500 nm.

In order to further understand the paired membranes induced by expression of IBV nsp4, electron tomography (ET) was used to visualize membrane rearrangements in three dimensions. In addition, ET was used to confirm that, unlike for other coronaviruses [26,38], co-expression of IBV nsp3 and 4 does not result in the formation of DMVs. DF1 cells were transfected with plasmids expressing either BeauR nsp4 or BeauR nsp4 with M41 nsp3. After 24 h, cells were fixed and processed for ET. The paired membranes produced by nsp4 expression (indicated by arrows) were found to form sheet-like structures with sections of paired membranes dilating in several places (arrowheads) (Figure 7a, Video S1). A comparison with cells expressing nsp3 and 4 showed there is no noticeable difference between the areas of paired membranes induced upon expression of these nsps (Figure 7b, Video S2). Therefore, expression of IBV nsp4 alone results in the formation of paired ER membranes. Addition of nsp3 does not alter the membrane structures induced with no formation of either DMVs, as seen for other CoVs or spherules.

## 4. Discussion

Induction of host cell membrane rearrangements is a tool used by many +RNA viruses, such as coronaviruses [1,2]. These membrane rearrangements vary between the different members of the family, with the *Alpha* and *Betacoronaviruses* inducing convoluted membranes and DMVs and the *Gammacoronavirus* IBV inducing zippered ER, spherules, and DMVs [19,20,21,22,23,25,26]. The formation of these membrane rearrangements is, however, a well-conserved mechanism used by these viruses in order to provide a site for viral RNA synthesis. Although the pool of knowledge about these structures has been growing, the mechanisms behind their formation remain largely unclear. Some light has been shed in recent years on the specific viral proteins involved in the formation of these structures; however, these studies were lacking in IBV. In this study, we looked at the involvement of nsps3, 4, and 6, which have all been implicated in the formation of membrane rearrangements. As transmembrane proteins, these are likely candidates in reordering the host cell membranes to the advantage of the virus. We showed firstly that DF1 cells are a suitable cell type to use for studying IBV membrane rearrangements in addition to those already tested [40].

In order to assess the involvement of nsps 3, 4, and 6 in virus-induced membrane rearrangements, plasmids expressing GFP, mCherry, or 3xFLAG fusion proteins were generated. To confirm expression of full-length fusion proteins, Western blots were performed using antibodies against the tags. For all the constructs, full-length nsp fusion proteins were detected. However, in cells expressing nsp3-GFP, an additional 49 kDa band was seen indicating that as well as full-length protein, a cleavage product corresponding to the C-terminus of nsp3 plus GFP was also being produced.

Next, we expressed nsps alone or in combination in DF1 cells to assess their ability to rearrange cellular membranes. When expressed alone, all three nsps had a reticular, cytoplasmic localization consistent with previous observations that these nsps localize to the ER [26,37,49,50,51,52,53], although nsp4 and nsp6 in addition had a punctate localization with nsp4 in particular forming large foci in some cells. ER localization was subsequently confirmed by colocalization of the three nsps with ER markers. When nsps3 and 4 were co-expressed, both proteins localized to large and small cytoplasmic puncta with some protein also remaining in the ER. This suggests that nsp3 and 4 are able to interact with one another, again consistent with previous findings for other coronaviruses [26,37], resulting in nsp3 moving into the nsp4-containing puncta. Co-expression of nsp3 and 6 or nsp4 and 6 did not result in alteration of their cellular localization. However, when nsp3, 4, and 6 were co-expressed, nsp3 and 4 colocalized as seen before but some puncta now also contained nsp6, although some puncta contained only nsp3 and 4 or nsp6 alone. This suggests that, as seen in other coronaviruses, nsp3 and 4 together, but not alone, are able to direct nsp6 into the nsp3/4 puncta [36,37].

Subsequently, EM was used to identify changes to the structure of cellular membranes upon expression of these three proteins. Surprisingly, expression of nsp3 did not induce any notable phenotype. Expression of nsp3 from either SARS-CoV or MERS-CoV results in the production of disordered membrane bodies likely derived from the ER [26,38]. It is not clear why nsp3 derived from IBV behaves so markedly differently from nsp3s expressed by other coronaviruses. However, the previously studied nsp3s have all been derived from *Betacoronaviruses* so nsp3 from *Gammacoronaviruses*, including IBV, may function somewhat differently. Indeed, an amino acid sequence comparison between nsp3 sequences from BeauR and the *Betacoronavirus* MHV A59 shows only 13.4% homology and 25.9% similarity. Therefore, although these are accepted as functional homologs, there is scope for these proteins to behave differently from one another. Furthermore, given that we have previously demonstrated that IBV-induced membrane rearrangements are distinct from those induced by *Alpha*- and *Betacoronaviruses* [25], differences in the mechanism of their formation might reasonably be expected.

Interestingly, expression of nsp6 alone induced membrane proliferation and the formation of disordered membranes similar to the disordered membrane bodies (DMBs) induced by SARS-CoV and MERS-CoV nsp3 [26,38]. Expression of nsp6 alone did not appear to induce microtubule organizing center vesiculation as seen upon expression of SARS-CoV nsp6 [38] and the presence of autophagosomes was also not apparent [49,54], although this is likely due to differences in experimental approaches, namely the use of EM in this study compared to immunofluorescence of whole cells used previously [54]. Therefore, IBV nsp6 also appears to function somewhat differently to nsp6 from SARS-CoV in its ability to rearrange membranes.

The most striking phenotype came upon expression of nsp4; expression of nsp4 alone was sufficient to induce areas of paired membranes. Furthermore, ET demonstrated that these are sheet-like areas of paired ER membranes, highly similar to zER in IBV-infected cells. It was noted that the paired membranes, although resembling zER in infected cells, lacked the electron density often surrounding the membranes [25]. This reflects the lack of the other viral proteins making up the replication complex, which, presumably, accumulate on the cytoplasmic surface of the zER. Nsp4-induced paired membranes were observed as both small regions throughout the cytoplasm and also in extensive areas of paired membranes. These two phenotypes potentially reflect the different localizations observed by confocal microscopy with some cells containing nsp4 localized only to the ER and some cells containing large cytoplasmic puncta corresponding to the large areas of paired membranes. Use of correlative light electron microscopy (CLEM) in the future would confirm this. Attempts were made to confirm the nsp4 homotypic interaction by co-immunoprecipitation; however, this was not successful. It has previously been shown for MHV that nsp4 can self-associate [37], although earlier attempts to demonstrate the interaction in SARS-CoV failed [55,56], highlighting that detection of this interaction can be challenging. However, it is likely that self-interaction between nsp4 proteins located in both membranes of the ER zippers the two ER membranes together to generate the paired membranes seen, although it cannot be ruled out that instead an interaction with one or more cellular proteins is required. Significantly, this is the first time for any coronavirus that, regardless of mechanism, a membrane pairing function for nsp4 alone has been described.

Surprisingly, addition of nsp3 did not alter the membrane rearrangements induced by nsp4 alone. Previous work by others has shown that for other related coronaviruses and arteriviruses, membrane pairing requires the expression of nsp3 and 4 (or their homologs) [36,37,38,57]. In addition to this, however, co-expression of nsp3 and 4 for other coronaviruses resulted in the formation of numerous DMV-like structures [26,38]. Despite extensive searching and the use of electron tomography to gain three-dimensional information, we were not able to detect any DMVs in cells expressing nsp3 and 4. The reason for this difference is not clear. Here, we used separate plasmids to express nsp3 and 4 but this strategy was also used in previous work and when compared with a cleavable nsp3–4 precursor did not yield different results [26]. Therefore, the protein expression strategy is unlikely to be the reason that DMVs were not formed. It is possible that the presence of the shorter nsp3 fragment detected by Western blot prevented the formation of DMVs. However, full-length nsp3 was also present and therefore should have been capable of inducing DMVs in combination with nsp4. In addition, DMVs were not detectable in cells expressing either nsp3 from M41 and nsp4 from BeauR or cells expressing nsp3 and 4 from M41, indicating that the use of proteins from different virus strains was not the reason for the lack of DMVs. Indeed, nsp3 relocalized to both BeauR and M41 nsp4-containing foci suggesting that M41 nsp3 is capable of interacting with both nsp4 proteins. Again, attempts were made to confirm interaction between nsp3 and nsp4 by co-immunoprecipitation, but this was not successful. Interactions between full-length or the C-terminus of nsp3 and nsp4 from other coronaviruses have been shown previously [37,55]. Interestingly, Sakai et al. showed that just two amino acid residues in nsp4 are necessary for the interaction with nsp3; however, these residues are only conserved in *Betacoronaviruses*, not in *Alpha*- or *Gammacoronaviruses* [58], so it is likely that the mechanism of any nsp3/nsp4 interaction is different in IBV. Overall, the data indicates that DMV formation by IBV requires the presence of additional viral protein(s), either to direct an interaction between nsp3 and nsp4 if it cannot occur directly or because DMV formation is via another mechanism. Co-expression of nsps 3, 4, and 6 did appear to result in the formation of a very small number of DMV-like structures. However, these were significantly less numerous and less easily identifiable than those observed by Oudshoorn et al. [26]. Therefore, nsp6 does not appear to be the IBV protein required, in addition to nsp3 and 4, to induce DMVs and other viral proteins must play a role.

Throughout this study, we were unable to detect spherules associated with IBV infection, although we did identify membranes highly similar to zER. In our previous work, we demonstrated that M41 virus has a low spherule phenotype and the region of the genome from the 5′ end to nsp13 was responsible for this [40]. Unfortunately, we were unable to clone nsp3 from BeauR due to toxicity problems in *Escherichia coli*. It was also not possible to clone nsp3 from two further strains of IBV. As the nsp3 used in this study was derived from M41, it is possible that this is the reason that spherules were not detected under any conditions. Nsp3 from BeauR and M41 are highly related with 90.5% amino acid homology and 95.2% similarity with the majority of the differences occurring within the non-functional papain-like protease 1 domain. Despite that fact, it cannot be ruled out that these differences are sufficient to prevent spherule formation. In future, cloning the C-terminal part of nsp3 from BeauR, as other groups have done for MHV [36], may provide further insight into the role of nsp3 in membrane modifications. An alternative explanation for the lack of spherules could be that the precise molar ratio of nsps to one another, as well as the presence of cleavage intermediates, generated as a result of expression via a polyprotein during virus infection is critical for spherule formation. In that case, the expression approach taken here of transfecting multiple plasmids into cells would not result in the correct ratio of proteins or presence of cleavage intermediates, thereby preventing spherule formation. However, Oudshoorn et al. were also unable to identify CMs and spherule-like structures when combinations of nsps were expressed either from separate plasmids or as a polyprotein [26]. Instead, it is more likely that additional viral proteins are required for spherule formation. This is not necessarily surprising. For *Alphaviruses*, spherules are only formed in the presence of all nsps and although they are able to form in the absence of RNA, the length of RNA present directly affects the size of the spherule produced [59,60]. Furthermore, in the case of Flock House Virus, spherules only form when RNA synthesis is actively occurring [61]. Therefore, spherule formation by IBV may require expression of additional nsps, including those required for RNA synthesis, as well as an RNA template. Alternatively, it may require expression of additional nsps that direct interaction with cellular proteins that facilitate changes to the membrane.

The mechanisms behind the formation of virus-induced membrane rearrangements required for replication organelle formation are doubtlessly complex. Although we have identified a clear role for IBV nsp4 in membrane pairing and the formation of zippered ER, numerous questions remain and further differences between IBV and members of the *Betacoronavirus* sub-family have been highlighted. The identity of the IBV proteins required for both spherule and DMV formation remain unknown and further study is required to complete our understanding of the critical stage of the virus replication cycle.

## Supplementary Materials

The following are available online at http://www.mdpi.com/1999-4915/10/9/477/s1, Table S1: Cells expressing each nsp compared to the total number of cells counted, Video S1: Electron tomographic reconstruction of paired membranes in an nsp4-expressing cell, Video S2: Electron tomographic reconstruction of paired membranes in an nsp3 and nsp4-expressing cell.

## References

[1] Miller S., Krijnse-Locker J. (2008). Modification of intracellular membrane structures for virus replication. Nat. Rev. Microbiol..

[2] Netherton C.L., Wileman T. (2011). Virus factories, double membrane vesicles and viroplasm generated in animal cells. Curr. Opin. Virol..

[3] Boon J.A.D., Ahlquist P. (2010). Organelle-Like Membrane Compartmentalization of Positive-Strand RNA Virus Replication Factories. Annu. Rev. Microbiol..

[4] Neufeldt C.J., Joyce M.A., Van Buuren N., Levin A., Kirkegaard K., Gale M., Tyrrell D.L.J., Wozniak R.W. (2016). The Hepatitis C Virus-Induced Membranous Web and Associated Nuclear Transport Machinery Limit Access of Pattern Recognition Receptors to Viral Replication Sites. PLoS Pathog..

[5] Harak C., Lohmann V. (2015). Ultrastructure of the replication sites of positive-strand RNA viruses. Virology.

[6] Paul D., Bartenschlager R. (2013). Architecture and biogenesis of plus-strand RNA virus replication factories. World J. Virol..

[7] Bienz K., Egger D., Pfister T., Troxler M. (1992). Structural and functional characterization of the poliovirus replication complex. J. Virol..

[8] Belov G.A., Nair V., Hansen B.T., Hoyt F.H., Fischer E.R., Ehrenfeld E. (2012). Complex Dynamic Development of Poliovirus Membranous Replication Complexes. J. Virol..

[9] Gosert R., Egger D., Lohmann V., Bartenschlager R., Blum H.E., Bienz K., Moradpour D. (2003). Identification of the Hepatitis C Virus RNA Replication Complex in Huh-7 Cells Harboring Subgenomic Replicons. J. Virol..

[10] Ferraris P., Beaumont E., Uzbekov R., Brand D., Gaillard J., Blanchard E., Roingeard P. (2013). Sequential biogenesis of host cell membrane rearrangements induced by hepatitis C virus infection. Cell. Mol. Life Sci..

[11] Doerflinger S.Y., Cortese M., Romero-Brey I., Menne Z., Tubiana T., Schenk C., White P.A., Bartenschlager R., Bressanelli S., Hansman G.S. (2017). Membrane alterations induced by nonstructural proteins of human norovirus. PLoS Pathog..

[12] Ávila-Pérez G., Rejas M.T., Rodríguez D. (2016). Ultrastructural characterization of membranous torovirus replication factories. Cell. Microbiol..

[13] Kujala P., Ikäheimonen A., Ehsani N., Vihinen H., Auvinen P., Kääriäinen L. (2001). Biogenesis of the Semliki Forest Virus RNA Replication Complex. J. Virol..

[14] Welsch S., Miller S., Romero-Brey I., Merz A., Bleck C.K.E., Walther P., Fuller S.D., Antony C., Krijnse-Locker J., Bartenschlager R. (2009). Composition and Three-Dimensional Architecture of the Dengue Virus Replication and Assembly Sites. Cell Host Microbe.

[15] Gillespie L.K., Hoenen A., Morgan G., Mackenzie J.M. (2010). The Endoplasmic Reticulum Provides the Membrane Platform for Biogenesis of the Flavivirus Replication Complex. J. Virol..

[16] Westaway E.G., Mackenzie J.M., Kenney M.T., Jones M.K., Khromykh A.A. (1997). Ultrastructure of Kunjin virus-infected cells: Colocalization of NS1 and NS3 with double-stranded RNA, and of NS2B with NS3, in virus-induced membrane structures. J. Virol..

[17] Cortese M., Goellner S., Acosta E.G., Neufeldt C.J., Oleksiuk O., Lampe M., Haselmann U., Funaya C., Schieber N., Ronchi P. (2017). Ultrastructural Characterization of Zika Virus Replication Factories. Cell Rep..

[18] Schwartz M., Chen J., Janda M., Sullivan M., den Boon J., Ahlquist P. (2002). A Positive-Strand RNA Virus Replication Complex Parallels Form and Function of Retrovirus Capsids. Mol. Cell.

[19] Snijder E.J., van der Meer Y., Zevenhoven-Dobbe J., Onderwater J.J.M., van der Meulen J., Koerten H.K., Mommaas A.M. (2006). Ultrastructure and Origin of Membrane Vesicles Associated with the Severe Acute Respiratory Syndrome Coronavirus Replication Complex. J. Virol..

[20] Goldsmith C.S., Tatti K.M., Ksiazek T.G., Rollin P.E., Comer J.A., Lee W.W., Rota P.A., Bankamp B., Bellini W.J., Zaki S.R. (2004). Ultrastructural Characterization of SARS Coronavirus. Emerg. Infect. Dis..

[21] Gosert R., Kanjanahaluethai A., Egger D., Bienz K., Baker S.C. (2002). RNA Replication of Mouse Hepatitis Virus Takes Place at Double-Membrane Vesicles. J. Virol..

[22] Ulasli M., Verheije M.H., de Haan C.A., Reggiori F. (2010). Qualitative and quantitative ultrastructural analysis of the membrane rearrangements induced by coronavirus. Cell. Microbiol..

[23] De Wilde A.H., Raj V.S., Oudshoorn D., Bestebroer T.M., van Nieuwkoop S., Limpens R.W.A.L., Posthuma C.C., van der Meer Y., Bárcena M., Haagmans B.L. (2013). MERS-coronavirus replication induces severe in vitro cytopathology and is strongly inhibited by cyclosporin A or interferon-α treatment. J. Gen. Virol..

[24] Zhou X., Cong Y., Veenendaal T., Klumperman J., Shi D., Mari M., Reggiori F. (2017). Ultrastructural Characterization of Membrane Rearrangements Induced by Porcine Epidemic Diarrhea Virus Infection. Viruses.

[25] Maier H.J., Hawes P.C., Cottam E.M., Mantell J., Verkade P., Monaghan P., Wileman T., Britton P. (2013). Infectious Bronchitis Virus Generates Spherules from Zippered Endoplasmic Reticulum Membranes. mBio.

[26] Oudshoorn D., Rijs K., Limpens R.W.A.L., Groen K., Koster A.J., Snijder E.J., Kikkert M., Bárcena M. (2017). Expression and Cleavage of Middle East Respiratory Syndrome Coronavirus nsp3-4 Polyprotein Induce the Formation of Double-Membrane Vesicles That Mimic Those Associated with Coronaviral RNA Replication. mBio.

[27] Neuman B.W. (2016). Bioinformatics and functional analyses of coronavirus nonstructural proteins involved in the formation of replicative organelles. Antiviral Res..

[28] Clementz M.A., Kanjanahaluethai A., O’Brien T.E., Baker S.C. (2008). Mutation in murine coronavirus replication protein nsp4 alters assembly of double membrane vesicles. Virology.

[29] Gadlage M.J., Sparks J.S., Beachboard D.C., Cox R.G., Doyle J.D., Stobart C.C., Denison M.R. (2010). Murine Hepatitis Virus Nonstructural Protein 4 Regulates Virus-Induced Membrane Modifications and Replication Complex Function. J. Virol..

[30] Beachboard D.C., Anderson-Daniels J.M., Denison M.R. (2015). Mutations across Murine Hepatitis Virus nsp4 Alter Virus Fitness and Membrane Modifications. J. Virol..

[31] Knoops K., Kikkert M., Worm S.H., Zevenhoven-Dobbe J.C., van der Meer Y., Koster A.J., Mommaas A.M., Snijder E.J. (2008). SARS-Coronavirus Replication Is Supported by a Reticulovesicular Network of Modified Endoplasmic Reticulum. PLoS Biol..

[32] Posthuma C.C., Pedersen K.W., Lu Z., Joosten R.G., Roos N., Zevenhoven-Dobbe J.C., Snijder E.J. (2008). Formation of the Arterivirus Replication/Transcription Complex: A Key Role for Nonstructural Protein 3 in the Remodeling of Intracellular Membranes. J. Virol..

[33] Pedersen K.W., van der Meer Y., Roos N., Snijder E.J. (1999). Open Reading Frame 1a-Encoded Subunits of the Arterivirus Replicase Induce Endoplasmic Reticulum-Derived Double-Membrane Vesicles Which Carry the Viral Replication Complex. J. Virol..

[34] Snijder E.J., van Tol H., Roos N., Pedersen K.W. (2001). Non-structural proteins 2 and 3 interact to modify host cell membranes during the formation of the arterivirus replication complex. J. Gen. Virol..

[35] Gorbalenya A.E., Enjuanes L., Ziebuhr J., Snijder E.J. (2006). Nidovirales: Evolving the largest RNA virus genome. Virus Res..

[36] Hagemeijer M.C., Monastyrska I., Griffith J., van der Sluijs P., Voortman J., van Bergen en Henegouwen P.M., Vonk A.M., Rottier P.J.M., Reggiori F., de Haan C.A.M. (2014). Membrane rearrangements mediated by coronavirus nonstructural proteins 3 and 4. Virology.

[37] Hagemeijer M.C., Ulasli M., Vonk A.M., Reggiori F., Rottier P.J.M., de Haan C.A.M. (2011). Mobility and Interactions of Coronavirus Nonstructural Protein 4. J. Virol..

[38] Angelini M.M., Akhlaghpour M., Neuman B.W., Buchmeier M.J. (2013). Severe Acute Respiratory Syndrome Coronavirus Nonstructural Proteins 3, 4, and 6 Induce Double-Membrane Vesicles. mBio.

[39] Lundin A., Dijkman R., Bergström T., Kann N., Adamiak B., Hannoun C., Kindler E., Jónsdóttir H.R., Muth D., Kint J. (2014). Targeting Membrane-Bound Viral RNA Synthesis Reveals Potent Inhibition of Diverse Coronaviruses Including the Middle East Respiratory Syndrome Virus. PLoS Pathog..

[40] Maier H.J., Neuman B.W., Bickerton E., Keep S.M., Alrashedi H., Hall R., Britton P. (2016). Extensive coronavirus-induced membrane rearrangements are not a determinant of pathogenicity. Sci. Rep..

[41] Casais R., Thiel V., Siddell S.G., Cavanagh D., Britton P. (2001). Reverse Genetics System for the Avian Coronavirus Infectious Bronchitis Virus. J. Virol..

[42] Darbyshire J.H., Rowell J.G., Cook J.K.A., Peters R.W. (1979). Taxonomic studies on strains of avian infectious bronchitis virus using neutralisation tests in tracheal organ cultures. Arch. Virol..

[43] Kremer J.R., Mastronarde D.N., McIntosh J.R. (1996). Computer Visualization of Three-Dimensional Image Data Using IMOD. J. Struct. Biol..

[44] Fang S.G., Shen S., Tay F.P.L., Liu D.X. (2005). Selection of and recombination between minor variants lead to the adaptation of an avian coronavirus to primate cells. Biochem. Biophys. Res. Commun..

[45] Promkuntod N., Wickramasinghe I.N.A., de Vrieze G., Gröne A., Verheije M.H. (2013). Contributions of the S2 spike ectodomain to attachment and host range of infectious bronchitis virus. Virus Res..

[46] Almazán F., González J.M., Pénzes Z., Izeta A., Calvo E., Plana-Durán J., Enjuanes L. (2000). Engineering the largest RNA virus genome as an infectious bacterial artificial chromosome. Proc. Natl. Acad. Sci. USA.

[47] Yount B., Curtis K.M., Baric R.S. (2000). Strategy for Systematic Assembly of Large RNA and DNA Genomes: Transmissible Gastroenteritis Virus Model. J. Virol..

[48] Yount B., Denison M.R., Weiss S.R., Baric R.S. (2002). Systematic Assembly of a Full-Length Infectious cDNA of Mouse Hepatitis Virus Strain A59. J. Virol..

[49] Cottam E.M., Maier H.J., Manifava M., Vaux L.C., Chandra-Schoenfelder P., Gerner W., Britton P., Ktistakis N.T., Wileman T. (2011). Coronavirus nsp6 proteins generate autophagosomes from the endoplasmic reticulum via an omegasome intermediate. Autophagy.

[50] Baliji S., Cammer S.A., Sobral B., Baker S.C. (2009). Detection of Nonstructural Protein 6 in Murine Coronavirus-Infected Cells and Analysis of the Transmembrane Topology by Using Bioinformatics and Molecular Approaches. J. Virol..

[51] Kanjanahaluethai A., Chen Z., Jukneliene D., Baker S.C. (2007). Membrane topology of murine coronavirus replicase nonstructural protein 3. Virology.

[52] Oostra M., Hagemeijer M.C., van Gent M., Bekker C.P.J., te Lintelo E.G., Rottier P.J.M., de Haan C.A.M. (2008). Topology and Membrane Anchoring of the Coronavirus Replication Complex: Not All Hydrophobic Domains of nsp3 and nsp6 Are Membrane Spanning. J. Virol..

[53] Oostra M., te Lintelo E.G., Deijs M., Verheije M.H., Rottier P.J.M., de Haan C.A.M. (2007). Localization and Membrane Topology of Coronavirus Nonstructural Protein 4: Involvement of the Early Secretory Pathway in Replication. J. Virol..

[54] Maier H.J., Cottam E.M., Stevenson-Leggett P., Wilkinson J.A., Harte C.J., Wileman T., Britton P. (2013). Visualizing the autophagy pathway in avian cells and its application to studying infectious bronchitis virus. Autophagy.

[55] Pan J.A., Peng X., Gao Y., Li Z., Lu X., Chen Y., Ishaq M., Liu D., DeDiego M.L., Enjuanes L. (2008). Genome-Wide Analysis of Protein-Protein Interactions and Involvement of Viral Proteins in SARS-CoV Replication. PLoS ONE.

[56] Von Brunn A., Teepe C., Simpson J.C., Pepperkok R., Friedel C.C., Zimmer R., Roberts R., Baric R., Haas J. (2007). Analysis of Intraviral Protein-Protein Interactions of the SARS Coronavirus ORFeome. PLoS ONE.

[57] Van der Hoeven B., Oudshoorn D., Koster A.J., Snijder E.J., Kikkert M., Bárcena M. (2016). Biogenesis and architecture of arterivirus replication organelles. Virus Res..

[58] Sakai Y., Kawachi K., Terada Y., Omori H., Matsuura Y., Kamitani W. (2017). Two-amino acids change in the nsp4 of SARS coronavirus abolishes viral replication. Virology.

[59] Kallio K., Hellström K., Balistreri G., Spuul P., Jokitalo E., Ahola T. (2013). Template RNA Length Determines the Size of Replication Complex Spherules for Semliki Forest Virus. J. Virol..

[60] Hellström K., Kallio K., Utt A., Quirin T., Jokitalo E., Merits A., Ahola T. (2017). Partially Uncleaved Alphavirus Replicase Forms Spherule Structures in the Presence and Absence of RNA Template. J. Virol..

[61] Kopek B.G., Settles E.W., Friesen P.D., Ahlquist P. (2010). Nodavirus-Induced Membrane Rearrangement in Replication Complex Assembly Requires Replicase Protein A, RNA Templates, and Polymerase Activity. J. Virol..

